# Supplementary material for: Healthcare system action on employment as a social determinant of health in people living with HIV: A qualitative study
Source: PLoS One. 2023 Apr 6;18(4):e0282421. doi: 10.1371/journal.pone.0282421 (PMC10079099; doi:10.1371/journal.pone.0282421)
Supplement: S1 Appendix — (DOCX) [file pone.0282421.s001.docx]

**Appendix 4G: Guide for HIV provider focus group**

**PREAMBLE**

Thank you all for participating in our focus group. During the next 45 minutes to an hour, we would like discuss your thoughts on employment in those living with HIV, barriers faced by this population, past experiences helping those with HIV gain employment and ideas on what a future intervention study might look like.

In terms of our definition for employment, we are referring to any paid work (full-time, part-time etc.)

First we will just go over some ground rules:

1. Respect is our number one rule –
   1. Please respect when others are speaking by taking turns and minimizing interruption
   2. Please respect that you all may have different ideas, experiences, perceptions and opinions – this adds to the richness of life and deepens our understanding
   3. Please respect the confidentiality of this discussion – you all know each other so we cannot keep your identity anonymous. Some of the information we share is for the benefit of all, but we ask that you use wise judgment and keep personal sharing confidential.
2. Pause times – some people will have longer pause times than others. If you are having trouble breaking into the conversation, please raise your hand so we can create space for you.
3. If you want to stop participating, you can simply stop participating.
4. Do you have any questions/comments before we start?

**QUESTIONS**

1. Can you tell me a bit about your practice and what populations that you primarily serve? (i.e. MSM, women, people who use drugs etc.) And about your roles on the team?

*refugee claimants – many people just learned their diagnosis.

What services to the ASOs?

1. Do you routinely discuss employment with your patients?
   1. Who generally brings up the topic?
   2. What is typically discussed?
   3. How do you document these conversations (in the medical record? How? Where?)
   4. What do you feel that your role is, if any, as a health care provider in helping PHA gain and maintain employment?
2. Can you tell me a bit about your patients living with HIV who are not working?
   1. Why do you think that they are not working? (i.e. issues related to HIV, needing to stay on disability to pay for medications, other health issues like substance abuse, mental health, or job market etc.)
      1. Why do you think that is a barrier to finding and maintaining work?
   2. Do your PHA patients who are not working generally discuss going back to work? (either desire/actually looking)
   3. Is this different from people living with other disabilities? What do you think is different with HIV?
   4. [only for FG in Ottawa] Anything specific about the context in Ottawa? Versus provincial or federal?
3. Can you tell me about your HIV positive patients who are working in any paid employment?
   1. How has HIV affected their ability to gain and maintain employment?
   2. Have you assisted with issues that they have faced at work? If so, how?

(i.e. sick days, time off for appointments, fear of disclosure etc.)

1. What are your thoughts on disability status (and access the ART) as a barrier to employment among PHA?
   1. What are your thoughts about the changing definition of disability for ODSP status?

(Aligning more with the federal definition which would limit it to those who have difficulty with speaking, hearing, walking, bowel or bladder elimination, feeding, dressing or mental function)

- - 1. How will this influence/affect your patients? In what ways?

1. Can you please describe your past experiences, if any, with helping your patients get employment? (i.e. disability accommodations, referrals to employment agencies or ASOs etc.)
   1. What information or resources do you think you might need to help your patients?
   2. Specific cases?
2. We are hoping to develop an intervention study to help PHA gain and maintain employment. (Give out handout with diagram from previous systematic review)

In a previous systematic review of employment interventions within a health care setting, we found that the effective programs included: 1) a multidisciplinary team of employment experts and health care providers that communicates regularly and collaborates, 2) a comprehensive package of services including expert advice, a job search, feedback, networking, education and training and peer mentorship 3) a one-on-one and tailored program depending on patient needs, 4) a holistic view of health and social needs and 5) prospective engagement with employers. We would aim to incorporate these aspects into our intervention study.

- 1. What are your thoughts about this type of intervention?
  2. Do you think this could be useful for your patients?
     1. Why or why not?
  3. Which of the above components do you think would be the most important to include in an intervention program with this population? Which are less important? Why? Is there anything missing?
  4. Would you refer your patients to an intervention study?
     1. Why or why not?
     2. Under what conditions would you refer? When would you not?

(patients, the study, the amount of time expected for the provider to be involved etc.)

- 1. Where would you see this being located? (i.e. in a clinic, ASO, community centre etc.)? Why?
  2. What methods do you think would be useful to recruit individuals?
  3. Format? (One-on-one? Group sessions?)
  4. Other comments?
