# Supplementary material for: Healthcare system action on employment as a social determinant of health in people living with HIV: A qualitative study
Source: PLoS One. 2023 Apr 6;18(4):e0282421. doi: 10.1371/journal.pone.0282421 (PMC10079099; doi:10.1371/journal.pone.0282421)
Supplement: Attachment — Submitted filename: Response-to-Reviewers.pdf [file pone.0282421.s003.pdf]

Andrew D. Pinto MD CCFP FRCPC MSc
CIHR Applied Public Health Research Chair
Director, Upstream Lab, MAP / Centre for Urban Health Solutions, Li Ka Shing Knowledge Institute, Unity Health Toronto
Physician, Department of Family & Comm. Medicine, St. Michael's Hospital
Associate Professor, Department of Family & Comm. Medicine, Faculty of Medicine, University of Toronto
Associate Professor (Status), Institute for Health Policy, Management and Evaluation
Associate Professor (Status), Dalla Lana School of Public Health, University of Toronto
Associate Director of Clinical Research, University of Toronto Practice-Based Research Network (UTOPIAN)

December 2, 2022

Re: Manuscript PONE-D-22-23683

Dear PLoS ONE Editorial Team,

Thank you for taking the time to review our manuscript entitled, *Healthcare system action on employment as a social determinant of health in people living with HIV: a qualitative study,* and provide us with feedback. We have addressed all editor and reviewer comments in the revised manuscript and think the paper is stronger as a result. Please see a detailed response to the comments below.

We look forward to hearing from you about this manuscript.

Sincerely,

Andrew D. Pinto MD CCFP FRCPC MSc

Comment	Response	Page
JOURNAL REQUIREMENTS		
Please ensure that your manuscript meets PLOS ONE's style requirements, including those for file naming. The PLOS ONE style templates can be found at https://journals.plos.org/plosone/s/file?id=wjVg/PLOSOne_formatting_sample_main_body.pdf and https://journals.plos.org/plosone/s/file?id=ba62/PLOSOne_formatting_sample_title_authors_affiliations.pdf.	We have updated the manuscript based on the PLOS ONE style requirements.	Throughout
Please ensure that you have specified (1) whether consent was informed and (2) what type you obtained (for instance, written or	The following has been added, "Written informed consent was obtained for in-	Page 8

verbal, and if verbal, how it was documented and witnessed). If your study included minors, state whether you obtained consent from parents or guardians. If the need for consent was waived by the ethics committee, please include this information.	person interviews and focus groups. Verbal informed consent was obtained using a verbal consent checklist completed by the facilitator for virtual interviews."	
We note that the grant information you provided in the 'Funding Information' and 'Financial Disclosure' sections do not match. When you resubmit, please ensure that you provide the correct grant numbers for the awards you received for your study in the 'Funding Information' section.	The Funding Information section only allows us to select one funder and input the number however in our financial disclosure we have put all co-author full funding statements.	N/A
We noted in your submission details that a portion of your manuscript may have been presented or published elsewhere. "The interview data for people living with HIV (PWLH) reported in this study have been used in a related manuscript published in the int. J. of Equity in Health, which focused on perceived barriers to employment from the perspective of PWLH. The manuscript we are submitting focuses on employment programs rather than barriers to employment, and includes significant additional data from health-care providers. Furthermore, the analysis for each paper was conducted separately, leading to a distinct set of themes focused on integration of health services and vocational rehabilitation. The related manuscript has been uploaded with this submission." Please clarify whether this publication was peer-reviewed and formally published. If this work was previously peer-reviewed and published, in the cover letter please provide the reason that this work does not constitute dual publication and should be included in the current manuscript.	The document that was uploaded with the submission was peer-reviewed and published in the International Journal for Equity in Health. As we noted in our cover letter, we do not believe that this constitutes dual publication because the focus of each paper is very different. The other manuscript focused only on barriers to employment from the perspective of PLWH. This manuscript focuses on employment programs and includes additional data from health care providers. Furthermore, the analysis for each paper was conducted separately, leading to a distinct set of themes focused on integration of health services and vocational rehabilitation.	N/A
Please review your reference list to ensure that it is complete and correct. If you have cited papers that have been retracted, please include the rationale for doing so in the	We have reviewed the reference list, and made minor adjustments to ensure citations are complete. No	Pages 24-27

manuscript text, or remove these references and replace them with relevant current references. Any changes to the reference list should be mentioned in the rebuttal letter that accompanies your revised manuscript. If you need to cite a retracted article, indicate the article's retracted status in the References list and also include a citation and full reference for the retraction notice.	articles cited have been retracted.	
EDITOR		
The abstract should include some information about how data was analyzed in the methods sub-section	Thank you for your comment. This information has been added.	Page 2
As reviewer 1 notes, please ensure to follow CORE-Q criteria for reporting qualitative studies and mention in limitations whenever certain CORE-Q aspects were not considered. Specifically, I would like to see some discussion of methodological orientation and theory (item no. 9) at the beginning of the methods section.	Thank you for this comment. We have added more details on the theoretical orientation of the study.	Page 7
REVIEWER 1		
Thank you for submitting this manuscript. While I enjoyed the content the scope of this review is to address the methodological evaluation of the submitted work. The team reported their methodology in the form of COREQ but didn't seem to consider how the aspects of recommended methodology not included in this design (e.g., not validation of findings with participants) can or should impact the study's conclusions. This lack of integration is a bit of a disconnect and once that could be addressed in the discussion. Further, identifying pts and providers different (i.e., city or clinic, some w/ age) is also inconsistent. I have no additional comments on the work's methodology.	Thank you for this comment. We have added a limitation to the discussion noting that we did not return transcripts to participants nor validate findings with them.<br><br>We did not identify specific providers in the transcripts of the focus groups therefore cannot present information on gender or age in the quotations. We believe that there is value to have this information remain in the manuscript despite the fact that it is different between the two groups. It is however consistent within the groups (of providers and patients).	Page 22

REVIEWER 2		
In the introduction, you note "However there are many barriers including uncertainty around losing disability benefits, particularly coverage for expensive antiretroviral therapy, and a fear of being trapped in a job to then maintain drug benefits (19,22–24)." Consider noting that these concerns vary by country, state/province. In the discussion you might want to come back to these concerns to comment on the fact that these drug coverage policies creating barriers to employment are political and can be changed, i.e., when you explain that in Ontario these concerns are unfounded (even though most people are unaware of this fact) you can use this as an opportunity to emphasize that in a context without ongoing pharmaceutical coverage for PLWH even once they are employed, employment assistance services really are not appropriate or feasible. Basically, Ontario has created the enabling policy environment so it can proceed to the next steps of raising awareness about this positive policy and then helping people actually access and navigate employment. But in the US, in places where coverage really would end if you got a job, you could not proceed with employment assistance.	Thank you for this comment. We have updated the manuscript accordingly to note that this policy is specific to Ontario and may not apply to other jurisdictions.	Pages 4 and 21
In the methods, a very brief description of the domains covered by the interview and FGD guide would be useful. I know you uploaded the guides as supplementary files but an in text summary would be helpful.	Thank you for this comment. This has been added.	Page 8
In table 1, please check the %. I.e. 2 participants in Ottawa were 18-25 years old… 2/20 should be 10% rather than 0%.	Thank you for the comment. This has been corrected.	Page 10
I think one area that could be highlighted in the discussion is that doctors have a role to play in explaining to patients that they can maintain their pharmaceutical coverage while going back to work. Doctors	Thank you for this comment. We have added this to the discussion.	Page 22

themselves were unaware of this program, so that strikes me as a key area for immediate intervention.		
You suggest that "Designing interventions to include employers could alleviate some uncertainties about repercussions of taking time off as a result of the episodic nature of HIV." It may be worth additional reflection on how this involvement of employers can or should be done in a manner that avoids disclosing employee HIV status or increasing stigma. Perhaps you could draw from literature on employment accommodation for people with other chronic conditions.	Thank you for this comment. We have added an addition reflection on avoiding forced disclosure when employers are involved.	Page 21
You note "Our study also found that PLWH suggest that health care providers should provide advice on the disclosure of status, as well as act as advocates with employers" – but was this a widespread sentiment? You may want to say that some PLHIV felt this way, but it seems likely that others did not want their doctor involved in their employment because they did not want emphasis placed on their HIV status. Is it a bit naïve to think that all employers will respond well to learning that their employee is living with HIV and that disclosure at the workplace is good for all employees?	Thank you for this comment. We have adjusted this sentence to say that health care providers could provide advice.	Page 21
You note "While there were some hesitations from both patients and providers, many of these can be addressed." You could further note that employment interventions integrated into healthcare settings would not be for all PLWH and thus could be there for those who want them and ignored by those who do not, rather than suggesting that with the right design these interventions could appeal to all PLWH.	Thank you for this comment. We have added that to the manuscript.	Page 22
